# Picturing healthcare: a photovoice study of how healthcare is experienced by service users in a mental-health low threshold service

**DOI:** 10.1186/s12913-022-08013-2

**Published:** 2022-05-30

**Authors:** Mariell Høgås, Toril Anne Elstad, Ottar Ness, Sissel Alsaker

**Affiliations:** 1 Department of Mental Health, Faculty of Medicine and Health Sciences, Norwegian University of Science and Technology, Trondheim, Norway; 2 Department of Education and Lifelong Learning, Faculty of Social and Educational Sciences, Norwegian University of Science and Technology, Trondheim, Norway

**Keywords:** Healthcare, Low threshold services (Lts), Recovery, Experiences, Documentation, Qualitative study, Photovoice

## Abstract

**Background:**

A recent policy change dictates that all mental healthcare in Norway must be referred and documented in the medical record of the service users. This has not been the case within low threshold mental health services, which is services without referrals, social arenas where healthcare professionals are available and where service users themselves can choose to attend based on their self-reported needs. This challenges the idea of “healthcare” being a medical term as opposed to experienced and expressed by the service user.

A new healthcare understanding that includes the service users’ voices are thus needed, and the aim of this study is to explore how service users within low threshold services, understand, describe, and experience healthcare.

**Methods:**

The present study has used the photovoice approach to explore how four service users perceive and experience healthcare in a low threshold context. The chosen photovoice method enabled service users to reflect upon and communicate their experiences first visually by individual pictures and thereafter reflective texts emerged through seven workshops. A qualitative thematic analysis was performed based on the pictures, notes and audiotaped material from the workshops.

**Results:**

The analysis found three main themes showing how healthcare is experienced: availability of people, availability of places and availability of activities. This is illustrated through the following sub-themes: First, common community, good relations and fellowship, second, flexible and easily accessible support, which includes the opportunity to spend time and to try things out in a safe environment, and third, facilitation and motivation for participation and activity, given the opportunity to have a meaningful role and be seen as a resourceful human being.

**Conclusions:**

A new understanding of healthcare is needed in the context of recovery-oriented low threshold services, as today`s largely medical understanding of healthcare is challenging to connect to a relational, recovery-oriented understanding of healthcare. As healthcare are to be documented in service users medical record, further research should explore how to document healthcare based on a new or extended, relational understanding of healthcare.

## Introduction and background

In 2017 a change occurred in low threshold services (Lts) within the mental health sector in Norway, as a new directive relating to documentation of healthcare in Lts were issued and created a policy change [1]. The directive lays down how laws and regulations are to be understood and practised and establishes that everything defined as healthcare at Lts needs to be documented in service users’ medical record. As up to that point in time some Lts had provided social support in the everyday life of service users, based on a recovery-oriented approach [2, 3], discussions about what is to be considered as healthcare in low threshold contexts arose. Healthcare is defined in the legislation. However, the reviewed literature lacks the voice of the service users who receive mental healthcare from the low threshold services when it comes to what and how they experience healthcare in these contexts.

Low threshold services are available arenas for people with mental health challenges, offering social, physical, and creative activities, without referrals, where healthcare professionals focus on facilitating meaning-making processes and managing everyday life [4–6].

Research has shown that mental-health Lts are experienced as good and supportive services [2, 4, 7] as they are available in the community and offer support to cope in everyday life.

As emphasised in official guidelines [8], the studied Lts employ a recovery-oriented approach with a focus on service users’ resources and on creating arenas for meaningful participation, coping and development according to each user’s own preferences, resources, and objectives [9, 10]. Over the last decade, both national and international guidelines have recommended a recovery-oriented approach within the mental health services [8, 11, 12]. Studies of a recovery orientation within the mental health services [13–16] have found that recovery-oriented Lts in the municipal mental health services provide a valuable service for the service users that complies with the national guidelines in force. However, what is considered as healthcare within these recovery-oriented low threshold services and who has the power of definition?

Legislation [17, 18] lays down the responsibility the local authorities have to offer preventive and health promotive services to people with mental health challenges. Lts like the ones in this study must address this and are now obliged to document the healthcare that has been provided. However, collaboration between professionals and service users within recovery-oriented services is based on a partnership where each person’s expertise is acknowledged and valued, regardless of roles [19]. This leads us to wonder how this partnership is affected by the mandatory documentation of healthcare and who decides what is and what is not healthcare. There is a need to explore how healthcare is experienced by service users in the context of recovery-oriented low threshold services.

To contribute to the discussion on healthcare and how to document it in service users medical record within these contexts, this study aims to explore how service users within low threshold services understand, describe, and experience healthcare.

## Structural and theoretical background

### Recovery orientation within low threshold services

Personal recovery is an internationally applied concept focusing on what people do to manage challenges to their mental health [20], both individually [21] and as a social process [22, 23]. Recovery-oriented low threshold services, like the meeting places studied in this article, aim to create meaningful content for the service users’ everyday life through these processes. This is in line with the literature that describes experiences of recovery as processual [24, 25], where support to facilitate access to resources in the context of mental health challenges needed to live a life in recovery in terms of being, doing and accessing [25].

The studied municipal mental-health low threshold services are meeting places in various local communities where people with mental health challenges can come on their own initiative to receive support, participate, and create meaningful content in their everyday life [2, 4, 16]. Service users and healthcare professionals at the studied low threshold services undertake a recovery-oriented mapping where the service users’ goals, resources and wishes for the use of the Lts are noted and commented on according to a resource-focused recovery orientation. Research has shown recovery-focused and strengths-based services to be central to improve opportunities for personal recovery [26]. This recovery-oriented mapping ensures that services within social arenas like meeting places, also is individually adapted, and thus will have many different approaches. The services provide assistance in the service users’ everyday life based on a recovery-oriented approach that provides support and opportunities for personal development in accordance with the service users’ own goals and resources. Healthcare professionals in such services convey hope and view people as experts on their own life situation. They use their professional competence to assist service users so they can expand their skills to cope in everyday life and to maintain and develop their social networks. An open and equal relation between healthcare professionals and service users is an important aspect within the recovery-oriented services [10, 27, 28].

For the service users, the core of a recovery-oriented practice underlines moving from “patient-centred help” to “person-centred help” [8]. This implies that the service users’ self-determination and personal empowerment should be a key part of their recovery processes, and that the individual is to find his/her own way forward. The Lts can provide and support partnerships between the service users and the services, which is a core recovery value [29].

According to Slade [30], many of the services working with mental health find it difficult to move on from traditional practice models towards recovery-oriented approaches. The essence of moving towards recovery is the change in the power aspect, as in recovery-oriented practice, the service user’s own expertise is given prominence [30]. It is therefore interesting to ask in this context whether a recovery-oriented and person-centred focus within low threshold community mental health services might be affected by the change towards mandatory healthcare documentation [1].

### Healthcare

The Ministry of Health and Care Services [1] has through directive 1–4/2017 instantiated the requirements for documentation of healthcare in Lts according to the provisions in the legislation. From this we see the need to clarify how “healthcare” is to be understood and defined in a low threshold context.

As the studied low threshold services are part of the local authority’s overall health and care service, and healthcare is offered by healthcare professionals, the legislation applies to them [17, 31]. According to the above-mentioned legislation, everyone who provides healthcare is obligated to document information about each service user. It is one thing to say that healthcare must be documented, but what is healthcare in a low threshold context? The studied Lts focus on supporting the service users in their individual recovery processes, instead of treating disease, and this service has traditionally been provided without referrals.

In the legislation, healthcare is defined as *all actions that have preventive, diagnostic, therapeutic, health preserving, rehabilitative or nursing and care purposes and that have been performed by health care professionals* [32]. This constitutes a structural, and medical definition of healthcare. In this study our aim is to explore how service users understand, describe, and experience healthcare. This is done through visual and verbal expressions from service users, focusing on how these experiences compares to today’s definition based on a medical understanding of healthcare and recovery-orientation within the services.

To manage the change towards mandatory documentation of healthcare in Lts a checklist was prepared by the local authority in the study [33]. The checklist presents four criteria that need to be present in the measurement for something to be defined as healthcare: The help must be 1) individually adapted, 2) based on personal information about the service user, 3) have a qualified action-oriented nature (for example when the user is asked by healthcare professionals to do something, or not do something, as a result of their dialogue) and 4) be part of the local authority’s health service [33].

The checklist further states that as the definition of the term healthcare is broad, it is necessary for healthcare professionals to make a clear overarching assessment in each case as to whether the help provided is healthcare or not, considering the purpose and how it is perceived by the service users.

As we can see, healthcare professionals use the checklist to assess and define something as healthcare. At the same time, the service users’ experiences have been described as important in such assessments [33]. This paper aims to contribute such experiences to this field.

## Methods

### Design

The aim of this study is to explore how service users within recovery-oriented mental health low threshold services understand, describe, and experience healthcare.

To explore and visualise service users’ experiences, a qualitative design, based on theories of human experiences and interpretation, was chosen for the study [34]. To capture the service users’ experiences, photovoice workshops were held at a low threshold service centre in a Norwegian municipality [35, 36]. Photovoice is an active and participatory method [36], which may empower those who find it challenging to express in words their experiences of healthcare. Further this method resonates with the recovery-orientation within the services, where service users’ active participation is central. Photovoice enables service users to communicate their experiences visually followed by verbal reflections.

All service users at two Lts in a Norwegian municipality received an open invitation to participate. Nine people in total signed up initially, these were both men and women. However, four men eventually attended the entire workshop process, and five participants dropped out. It was a coincidence that only men joined the whole process. These participants have a long history of mental health issues and have been service users at the low threshold services for five years or more. They are men well spread in the age range between 33 and 69 years old, living in own apartments. One of the participants (B) receive additional individual mental healthcare from the municipality, while the other three participants (A, C, D) self-report that they manage their everyday life living with mental health challenges, much based on the support they receive at the low threshold service.

In total, seven workshops were held during a period of four months facilitated by the first author (see Table 1). The pictures and text material from the photovoice workshops were examined according to a four-step reflexive thematic analysis as described by Johannessen, Rafoss and Rasmussen [37], which is further adapted from Braun and Clarke`s [38] six step thematic analysis.

**Table 1:** How the workshops unfolded

	Workshop 1	Workshop 2	Workshop 3	Workshop 4	Workshop 5	Workshop 6	Workshop 7
Theme	Welcome Study aim What is photovoice? Photo-exercise Take photos individually until next workshop Lunch	Study aim Repetition: What is photovoice? Transfer digital photos Composition Take photos individually until next workshop Lunch	Transfer digital photos Reflection with pictures on the floor: The participants talked about own pictures, and the group gave further comments to each other’s pictures Use of audio recorder Lunch	Reflection with pictures on the floor The participants talked about own pictures, and the group gave further comments to each other’s pictures Use of audio recorder Lunch: Pizza	Work with text related to pictures Each participant could choose 1–8 of their pictures to write text related to Lunch	Work with text related to pictures Lunch	Preparing the photovoice exhibition In total 28 pictures and texts were completed
Time	4 h	4 h	4 h	4 h	4 h	4 h	2 h
Participants attended	A, B, C, D + first author	A, B, C, D, E + first author	A, B, C, D + first author	A, B, C, D + first author + last author + 2 healthcare professionals	A, C, D + first author	A, B, C, D + first author	A, B, C, D + first author

.

### Data generation from the workshops

The research material consisted of 28 pictures with accompanying texts and participants audiotaped reflections that related to the photo-expressions from the workshops. Additionally, the first author made notes after the encounters, consisting of descriptions of the workshops, and immediate thoughts and reflections of the workshop-situations. Further the notes consisted of practical information as how many showed up, what were the plans for this workshop, how did the participants engage during the workshop, and thoughts about how to take (develop) this process further. A rich narrative material emerged and grounded the analysis together with background material from the health service studied. This is data material that require interpretation in context.

In more detail data was generated as follows (se also Table 1). As a first step, the research project was presented at house meetings at the two low threshold services, where the projects aim, design and ethics in relation to confidentiality and the opportunity to withdraw from the study were presented. Participants interested in the project signed the informed consent form. Seven photovoice workshops were held in the facilities of a mental-health low-threshold service in a Norwegian city.

At the first workshop the photovoice method were described to the participants, in addition a movie was used to show how the photovoice method could be used to explore a research question. The participants seemed excited to use photo in such a way. At the same time, they expressed concern related to how they could communicate what they wanted to say through this. The challenge for the first author was to avoid giving too many guidelines related to the method, but instead enable the service users to try out themselves. A photo exercise was arranged for the participants followed by reflections among the group members. After this experience the assignment related to this study were presented for the group: to take individual pictures of what they experienced as healthcare in Lts and bring to the following workshops. On the second and third workshop the digital pictures were transferred and printed on paper. Further on the third workshop, the group, including the first author, started to reflect on the pictures. All the pictures were put on the floor, and the group sat on chairs in a circle around the photos and commented and reflected upon own and others’ photo expressions. The four men were asked to talk freely about what they wanted to communicate through their pictures, and to add their reflections on each other’s pictures. No further guidance was presented. The participants therefor talked about own pictures, and the group gave further comments to each other’s pictures, based on own experiences of healthcare in this context, that was retrieved and made visible to them through watching the pictures. On the fourth workshop the same reflection approach was used, and in addition the last author and two healthcare professionals participated in the reflection of the pictures, together with the group and the first author. The healthcare professionals were asked to participate and listen to what the service users told, and if possible, ask follow-up questions, to enrich the reflections. The purpose of bringing in more people to listen to the reflections and provide input, was also to open the opportunity for the participants to talk more freely about what they wanted to communicate through the pictures about healthcare, to someone who had not participated from the beginning. Furthermore, the intention was to create a space for service users and healthcare professionals to reflect upon service users` experienced healthcare in a low threshold context. Maybe this could inspire a common further focus on healthcare in such a context, as the focus on healthcare in legal terms is new for these services.

The reflections were audio-recorded on the third and fourth workshop and later transcribed by the first author, who also took notes along the way, referring to subjects that was focused on related to healthcare in the reflections. Transcriptions for each picture (collected excerpts) from the conversations/reflections, as well as the first author’s notes on each picture from these reflections, were copied, and handed out to the participants to whom the different pictures belonged. This involved both their own and the group’s comments from the conversations. During workshop five and six the participants worked with the text individually, as well as in the group. The four men did not tell coherent stories but talked about different episodes and aspects of experienced healthcare. Through this process they expanded each other’s ideas related to the pictures, as the pictures triggered different experiences for the participants. Each participant could choose 1–8 of their pictures to write text related to, which resulted in 28 pictures with related texts. The picture’s owners decided what text/excerpts they wanted to use to represent experiences of healthcare in the low threshold context. In this writing process the participants had control over the text created to the pictures. Further they decided if they wanted to add more text or write their text into a coherent whole. This formed the groups expressions about healthcare. Through this process we move from individual contributions to the group’s expressions. These pictures and texts would then be part of a photovoice exhibition, and further serve as data material for this study. This generated 28 pictures and 28 accompanying texts. There was an ongoing process among the service users that created the pictures and texts. During this process the service users did an analysis which resulted in the pictures and text representing experienced healthcare, presented in the exhibition. This process is not a part of our analysis but serve as data generation. We have chosen to use the data material from the photovoice exhibition in our further data analysis.

### Data analysis

During the photovoice workshops the service users at the low threshold services presented narrative material based on their experiences of mental healthcare in this context. The participants presented their own pictures of healthcare and developed accompanying texts in the group (see data generating). Notes of more unstructured conversations and reflections during the workshops were made by the first author, in addition reflections from the group on the third and fourth workshop were audiotaped and transcribed by the first author. These transcriptions and notes were handed out to the participants, so they could work further with writing texts to their pictures, based on how they wanted to make visible experienced healthcare. This made the groups expressions visible, still chosen individually by the picture’s owner.

In this article we have used the material from the time the 28 pictures and the 28 accompanying texts representing the groups expressions of healthcare were present, as well as background information, to form the basis for a qualitative thematic analysis inspired by Johannessen et al. [37], using the following steps:

1. Preparation, by gathering the pictures and accompanying texts created by the group.
2. Encoding: The first author reviewed each picture with accompanying text from the participants and then strived to point out statements in the material expressing the experience of healthcare. In addition, background information and the first author`s notes from the workshop situations where she was part of the reflective process influenced the further analysis and interpretations.
3. Categorization: After pointed out statements of experienced healthcare in the material as step two, the first and the last author sorted out some common themes in the material and categorised the 28 pictures/texts with short statements describing the service users’ experiences of healthcare in the low threshold context from the original texts, under relevant theme names. Six sub-themes were generated in this way, structured by direct quotes from the participants. The six sub-themes were then further developed by the first and the last author into three main themes, which comprised the overarching theme availability.
4. Reporting and writing of the findings: The first author selected from among the 28 pictures in the data material and chose one picture that nourished the analysis, to represent each theme. An excerpt from the text material created by the participants was presented in italics next to each presented picture, before moving on to the discussion part.

The second and third author participated in discussions together with the first and last author during the project process, as well as readings, feedback sessions and reflections on drafts in working towards the final manuscript. All the authors took part in reading and commenting on the article draft during the writing process. The analysis, showing the service users’ expressions and experiences of mental healthcare, provides valuable insight; our findings show the availability of people, places, and activities to be experiences of mental healthcare in the low threshold context. Finally, a poem structured by the first author, based on the participants texts of experienced healthcare, provide an alternative summary of the findings. We chose to use this alternative way of presenting the main content, as we believe that this way of promoting the findings could be useful for the reader, to clarify the content.

### Methodological considerations

The participants in the present study, four men, were recruited from low threshold services where the first author had upfront context knowledge, through having worked in low threshold services over many years. It could serve as a plus that the first author knew the context and therefor did not have to use time to get a context overview. On the other hand, this upfront knowledge could make one look blindly at new information. The study therefor focused on making the service users voices central and visible through pictures and texts, using the photovoice method. The pictures gave access to the service users’ experiences and prompted associations, which was a different and engaging way to gain access to the service user’s voices. We could have used interviews as our method, but we chose an active participatory approach with pictures from the context, a non-verbal approach, as the foundation for further conversations and reflections.

Potentially the first authors upfront knowledge could have had an impact on the service user’s participation in this study and how they answered any questions. Recruitment was therefore arranged through healthcare professionals at the Lts – information about the study was provided at house meetings, interested service users then notified the healthcare professionals who then contacted the first author.

The first author took part in reflections during the photovoice workshops with a clear aim and focus on the service users’ expressions, both in pictures and texts. Multiple lead-ins were used to clarify the service users’ voices and what was the important meaning for them. Furthermore, the photos provide possibilities – showing experiences in the specific context, and prompt associations. The studied photos unite the service users’ voices with structural voices.

All authors are skilled within mental healthcare, from different professions; social work, occupational therapy, nursing and educational psychology, and brought their scholarly as well as their clinical experience from mental health work into the analysis. The author-team has a varied background with both academic and practice-related education, which provides a good understanding of the context. At the same time, it might be a limitation because one might be biased. Based on these limitations it was important to have focus on the service users’ voices during the whole process.

The findings in this study are from a concrete service in a municipality in Norway, and there will be limitations in relation to a more general expression. At the same time – in the discussion we have highlighted literature from similar studies, which helps to express and develop the field that we discuss. The findings do not seek to be generalizable, but rather to find unique entrances to develop nuances and possible understandings related to the term “healthcare,” based on service user’s experiences within the low threshold context. To do this, we put our findings into communication with existing knowledge in the field.

## Ethical considerations

The project has been reported to and approved by the Norwegian Centre for Research Data (NSD) with project reference number: 983790. The project was also assessed by the regional committee for medical and health research ethics (no. 2018/1993/REK midt) and found acceptable as the purpose of the study is not to obtain new knowledge about health and disease, but rather to explore service users’ experiences of the services. The staff at the services informed about the project at a house meeting and handed out a letter of information. The first author then was invited to come to the low threshold service and inform about the project to those who were interested. The participants were informed about the project plan, the aim of the study, how anonymity would be protected and the right to withdraw from the project at any time during the research process without any consequences. As mentioned above, all the participants signed a written consent form before the first photovoice workshop. This article aims to make the service users’ voices heard with respect to the development of a new healthcare understanding in the low threshold context. We maintain that it is important to include the service users’ voices in this development and to make them equal partners in the process.

## Findings / results

The availability of possible resources such as other people, safe places like meeting places and different activities, in the everyday life of service users is the main finding when it comes to how mental healthcare is practised and experienced in this low threshold mental health context. Availability is a main point in our material and the poem below, which emerged from the data material, are structured by the first author inspired by quotes from the participants related to experienced healthcare, after finalised the findings and serves as a summary and a frame for our findings in this paper. Our analysis provided three main themes referring to how mental healthcare is experienced and linked to availability: (main theme 1) availability of people, (main theme 2) availability of places and (main theme 3) availability of activities. In the following section we will present the main themes and sub-themes followed by analysis and discussion. The findings are first presented schematically in Table 2 to gain an overview of the themes and sub themes, before pictures and texts from the photovoice workshop are presented, together with a short, first reflection from the first author. Finally, a poem structured by the first author sum up the findings section.

**Table 2:** Overview of main findings

Main theme 1: Availability of people
**Sub-themes:** **1.1** Common community, good relations and fellowship are healthcare Quote: *It means something to be one of those who sit around the fire (…)* **1.2** Flexible and easily accessible support is healthcare. Quote: *In the low threshold service, you’re not caught in these tight lines and processes but have the freedom to choose (…)*
**Main theme 2: Availability of places**
**Sub-themes:** **2.1** To come here, is healthcare Quote: *I do it to help myself now. To me it’s healthcare to come here (…)* **2.2** Opportunity to spend time here, and to try out things in a safe environment is healthcare Quote: *At the low threshold service you can spend the time you need to find the right key (…)*
**Main theme 3: Availability of activity**
**Sub-themes:** **3.1** Facilitation and motivation for participation and activity is healthcare Quote: *I was able to try out new activities where I discovered latent talents and that helped me to grow (…)* **3.2** Given the opportunity to have a meaningful role and to be seen as a resourceful human being is healthcare. Quote: *I’m doing something I like, at the same time as I’m pleasing others (…)*

### Main theme 1: availability of people

#### Sub-theme 1.1: common community, good relations and fellowship are healthcare

A picture from the workshops, and participants expressions related to the picture:



This image and text show the value of coming together and belonging to a community. This provides service users with someone to talk to, someone to do things together with, but it also helps to avoid being alone. To us the bonfire symbolises light and warmth, where people see each other, create relations, share and receive support. The expressions also show the value of availability of social connections in everyday life. The cooperation needed for the bonfire to catch fire may also help to create fellowship and warmth.

### Sub-theme 1.2: flexible and easily accessible support is healthcare

A picture from the workshops, and participants expressions related to the pictures:



The statement “small village” embraces close relations, and people knowing and supporting each other. The availability of people, the loose frames, the freedom to choose and the power to seek support from others on one’s own terms, are interpreted as valuable. An open framework like this might empower people to use their autonomy and resources to take responsibility, seek help from others when needed and build a common community with others.

### Main theme 2: availability of places

#### Sub-theme 2.1: to come here is healthcare

A picture from the workshops, and participants expressions related to the pictures:



We find that it is valuable to have an available, supportive service in the community, a place to go to and spend time with others during one’s everyday life, a social arena. The service might have health-preventive and health-preserving qualities as it helps people to create everyday life routines, stability, and creates recovery with meaningful content in life when otherwise feeling fear and teetering on the edge.

#### Sub-theme 2.2: opportunity to spend time, and to try things out in a safe environment is healthcare

A picture from the workshops, and participants expressions related to the pictures:



The findings show that it is valuable to have a service that makes different opportunities available and gives the autonomy to choose, where there is no predetermined path. Perhaps one key fits, or perhaps there are many suitable keys, interpreted as possibilities in this context. Spending time with others in a safe environment is found to be an important part of seeking the right individual path.

### Main theme 3: availability of activity

#### Sub-theme 3.1: facilitation and motivation for participation and activity is healthcare

A picture from the workshops, and participants expressions related to the pictures:



The findings show that low threshold services can serve a valuable role by making possibilities available, offering an arena where service users can participate and then discover their own resources, where they can try out things, experience mastering something new and where they can work together with others on these activities.

#### Sub-theme 3.2: given the opportunity to have a meaningful role and be seen as a resourceful human being

A picture from the workshops, and participants expressions related to the pictures:



The findings show that the studied low threshold services facilitate for mastery through activity by making multiple activities available. Each person counts and every user has the opportunity to create a valued and meaningful role for themselves through active participation and contribution with peers. The findings also show that the availability of activities can contribute to service users’ recovery processes, as participation within safe environments might be the first step towards extended participation on a wider scale in life.

### Findings summed up by a poem structured by the first author:



## Discussion

### Main theme 1: availability of people

One of the challenges when struggling with mental health issues relates both to developing relations and maintaining them over time. Social arenas such as the low threshold services (Lts) provide opportunities where supportive relationships like this can be forged and maintained. The question is whether healthcare is provided at these places. Our findings show that the availability of people is seen as healthcare for the participants in this study. In Lts, both healthcare professionals and peers are available, giving the opportunity to be part of a larger context. The Lts offer easy access to healthcare professionals and the relevant literature finds their role to be important [6, 39, 40]. This enables healthcare professionals to support the service users over time, and to inspire hope, described as an important task within recovery-oriented services [14]. Service users of services relevant for personal recovery have described that ongoing personal relationships contribute to experiencing continuous care [41]. In a study about acute day units’ informal interactions with peers and staff are described as important for recovery from mental health challenges/crises [42]. Peers and staff being available for interactions might through this contribute to personal recovery for the individual. At the Lts in this study, people are available so that relationships are established and last over time.

The participants said *it means something to be one of those who sit around the fire (…).* We find here that it is meaningful to be part of a common community instead of being alone in the dark. This is in line with studies that have shown that day centres give service users a feeling of cohesion [43, 44]. Spending time together with others may create valuable relations that might also develop into healing relationships. Social connections at the Lts are found to be valuable throughout our analysis, in the same way as Borg and Topor [27] describe humans as social beings who develop themselves and relationships by spending time with others.

From our analysis, the bonfire photo in our findings is an example of this. The participants looked at this photo during the workshops and talked about the outdoor trips facilitated by the Lts, where each group member had the opportunity to contribute their resources. We find that this type of participation and cooperation makes the service users’ resources visible and contributes to the forming of relations and solidarity, and for them this was what healthcare was all about.

The participants in our study underline the importance of the freedom to choose within the Lts and they picture flexible services as healthcare. The researchers interpret flexibility to mean being able to come and see and talk to people when it suits them, as well as being able to choose who to talk to. This type of flexibility is recommended in the literature when it comes to recovery-oriented support [19]. Furthermore, the autonomy this flexibility provides is also experienced as fundamental support for personal recovery [19].

Our findings show that the Lts are social arenas where people are available that the service users can choose to attend. The participants refer to a sense of community at the Lts where they are experienced *almost like a small village.* Put in the context of the second picture of the big city with tight lines and no people, society at large, Lts might be understood as small communities of people that can provide safe relations within the greater society. This can represent stability at a time when other aspects of one’s everyday life seem unstable and difficult.

Our findings show that to manage personal recovery processes, people need to be around others and to support each other. This is supported by Reed, Josephsson and Alsaker [3], who found that recovery unfolds as collective processes by doing everyday activities together with others. As the African proverb says, “it takes a village to raise a child” [45], we argue that “it takes a community to support a person’s recovery processes”. The proverb is a metaphor for the idea of encouraging additional community involvement to achieve growth and development [45]. Our findings show the value and the energy created as people come together, work together, and feel a sense of community, of fellowship, through the warmth spreading from the fire. If people in the smaller community come together to support each other in their recovery processes, the safe framework might expand to include larger parts of the community, giving today’s service users of Lts even more flexibility, a larger network and more availability.

When it comes to the question of whether or not healthcare can be defined as facilitating social gatherings and ensuring that people are available within the framework of Lts, our findings point in this direction, in line with Sæterstrand and Møllersen [46], who showed that the most important task for nurses in a day centre was to facilitate for social togetherness. While we have found this to be a fundamental underpinning of healthcare at the lts, the traditional understanding of healthcare might find this to be challenging.

Summing up the findings from this main theme one might ask that if the availability of people providing support is expanded to reach beyond the low threshold service framework, how will it be defined under the healthcare term? Which actions and relations would then be considered as healthcare?

Low threshold services provide opportunities to spend time with others, focusing on both personal and social recovery processes. The literature, e.g., Ness, Borg and Davidson [15] finds that supportive social relationships are facilitators of recovery. As community mental health services are aiming for a recovery orientation, it is important to discuss which place healthcare should have in this, and how we can connect the two concepts.

Being around others helps one to gain new insights and the motivation to act and progress in the recovery process. Reed, Josephsson and Alsaker [3] have also found this, showing that recovery unfolds as unique and collective processes rather than as individual processes.

### Main theme 2: availability of places

A city of some size might have many mental health services dedicated to people with challenges. In the referral-based services, healthcare professionals reserve the right to assess who is to receive healthcare, while in low threshold services like the studied meeting places, the service users can choose to use the service based on their self-defined needs [16]. One of the participants said *(…) In the low threshold service you’re not caught in these tight lines and processes but have the freedom to choose. Flexible healthcare (…).* It is thus found to be valuable when people have the flexibility to choose if and when they want to attend such places as Lts, and if they want to opt out of the service altogether. Flexible healthcare, as sited in our findings, is understood in this context as places where healthcare is available.

Our findings point out that having a place to go when the need presents itself and being aware of this possibility play an important role in one’s everyday life and serve as healthcare for the service users in the studied Lts. The literature describes Lts as available places in local communities that are both welcoming and provide the opportunity to spend time in a safe environment [3]. The studied Lts have a recovery-oriented approach that enables the service users to have an active and participatory role in the facilitation of these places [29]. Other researchers have also pointed out the importance of the facilitation concept within services and centres like Lts [39, 47].

Our findings show that by providing the possibility for people to come and create a meaningful everyday life, the Lts contribute to the personal recovery process. Cocchi and DeIsabella [47] point out the need to have a broad perspective on facilitating meeting places, as they address different needs within people’s everyday lives that might be challenging to manage in society at large. In a review study by Bachke and Larsen [48], day centres are presented as good integrating places outside the greater society, and they highlight their function of protecting service users from society, while also problematising whether this might inhibit real integration in society [48]. As our findings refer to Lts as safe platforms, we interpret them to be the first step towards a greater degree of integration into society at large.

In our findings one of the participants said, *I get healthcare at the low threshold centre when I need it.* This concurs with Bachke [39], who maintains that having staff available at a Norwegian meeting place underlines the importance of being there and offering support when the user is ready for it. Rise, Westerlund, Bjørgen, and Steinsbekk [49] also advise healthcare professionals to provide flexible support. Bearing all this in mind, we find that it is important that the framework for places like Lts is visible in the community so that people who need healthcare will see the health-promoting effect such centres have. Places that are welcoming and promote participation in the community are found to contribute to health and well-being for people struggling with their mental health [50].

In our finding’s healthcare is referred to as having a place like the Lts where you can come, spend time, and try things out in a safe environment. One of the participants said (…) *At the low threshold service you can spend the time you need to find the right key.* The findings show that the service user’s autonomy is an important aspect in the studied Lts, where having time to spend in a safe environment is a key element.

For some, just coming to the Lts is enough. One of the participants said: *to me it’s healthcare to come here*. This is in line with recovery-oriented values that refer to people as powerful and resourceful. Places such as Lts enable people to use their resources, first to come to these places and then to participate there. One of the participants said, (…) *after a while I was confident enough to try things on my own.* Places like Lts facilitate ongoing processes according to individual needs. This is supported by Brandal, Bratberg and Thorsen [51] who find that it is important for health services to be available according to specific needs. But who has the power to define these needs? When in recovery, the user’s own defined goals are important, and there is no set, predetermined path. As the picture of all the keys suggests, there can be many suitable keys, meaning many possible paths, which in turn means that openness and availability are core elements in recovery-oriented Lts.

The legislation defines healthcare as actions taken by healthcare professionals that have health-preserving and rehabilitation purposes [32]. Our findings present a wider understanding of healthcare in terms of facilitating Lts as a healthcare framework. Our findings support this: the service users need time to work towards their goals and to conduct rehabilitation actions as they participate at their own tempo.

As recovery is a collective and personal process [3], we interpret that the framework of a social arena like Lts contributes good support from both healthcare professionals and peers that in turn serves as healthcare. Familiarity with Lts and being willing to attend them can offer an activity in the service users’ everyday life in addition to routines, stability and a safe platform, something our next finding will elaborate on. Considering this, we wonder if it is possible that the framework making Lts available is in itself a health-promoting measure.

### Main theme 3: availability of activity

Our findings show that doing activities together has great value; when people do something they like, they learn new things and share the activity with others. In a historical review of day centres in Great Britain, Bryant [52] found that the socialising aspect of the activities aids recovery. Furthermore, participation and contribution in ordinary everyday activities within social contexts where the service users feel valued is described as “recovery as doing” by Sommer et al. [25]. The researchers find that activities like this have a health-preserving function, which is defined as healthcare in the legislation.

Healthcare professionals might facilitate activities within low threshold services, but our findings show that motivating service users to participate in activities might be equally important. Eklund and Sandlund [53] point to the importance of having opportunities to choose an activity when motivating service users. Our findings have shown possible activities within the service, where the motivation to take part might come from multiple angles. As one of the participants said, *At the painting group we paint together, we work together and are motivated and help each other with tips.* This togetherness reveals that a fellowship is created through participation in activities. Moreover, when participating in activities at Lts, new resources might surface. One of the participants said: (…) *I was able to out try new activities where I discovered latent talents and that helped me to grow. This has been healthcare for me as it has opened a new world to me.* The researchers understand that the activity contributes to movement within personal processes, as referred to in recovery-oriented practices [3] and supports social processes. Supporting such participation in meaningful activities is described as significant within recovery-oriented services [14]. Further, research show that meaningful activities is a valued feature within mental health [42], as it contributes to a sense of safety, making structure through activity. Furthermore, valued everyday activities for people attending meeting places are described in literature as encouraging social engagement and providing room to be creative and learn new things [54]. One participant said (…): *I have participated in a painting exhibition with my paintings, facilitated by the low threshold service, and I sold some pictures (…).* Activities like this might contribute to a considerable degree outside the services as well. Larsen [55] supports this by seeing day-centre activities as a stepping-stone into society for some, while for others they are meaningful while they are attending the Lts. Our findings show that the painting activity at the studied Lts can be seen as a multi-layered contribution in a person’s everyday life. Such contributions are a) the concrete painting activity itself, b) spending time with others, c) sharing ideas, d) working on a product and finishing it, e) sharing the result with others and e) the powerful feeling of adding something valuable, such as a painting. This is also a recovery process providing individually adjusted and flexible support negotiated by several contributors [16], in this case Lts, healthcare professionals, peers, community members and others.

In our findings one of the participants said: *baking is an activity I master and that contributes to my health. It makes me stronger. I work together with the staff, and they motivate me.* Healthcare professionals motivating and supporting service users in a concrete activity is described in the literature as an important recovery-oriented service [14]. The participant draws attention to the value of doing an activity, and when it is mastered, it is a valuable contribution to this user’s health. At the same time, working together with others substantiates the principle of equality that is a key part of recovery orientation [8, 27]. Doing activities together, regardless of the roles at the low threshold centre, evens out any differences as the focus is on the activity itself. Furthermore, our findings show that when people do activities, they know they manage and enjoy doing, a feeling of joy is created, and even greater joy is experienced from sharing the activity with others. Engagement in different activities makes meaningful social roles visible and is described by Snethen et al. [50] as a desirable feature and important opportunity within community locations.

Activities described in our findings, such as painting and baking, are two out of many possible activities found at the studied Lts. For some people it is sufficient to have an activity to come to in the Lts. Service users interviewed by Elstad [2] described coming to the centre during difficult times as a type of mastering. The availability of activities at the low threshold services adds value and opportunities to people’s lives.

A question that arises is whether such activities are considered as healthcare, and if so in which context they are considered as such. Finally, will the same activity automatically function as healthcare for everyone attending?

## Closing comments / conclusions

Our findings show many aspects that comprise healthcare: availability of a common community, good relations, fellowship, flexible and accessible support, a place to come to, a place where one can spend time and try out things in a safe environment, where one can be motivated to participate, facilitation of activities and having a meaningful role. All these available aspects are important in many service users’ everyday life settings. The studied low threshold services are shown here to be everyday life settings for people living with mental health challenges.

Day centres have changed from being dominated by treatment ideology, as in the 1960s (medical understanding), to being dominated more by recovery-oriented approaches, as described by Bachke and Larsen [48]. Low threshold services, such as meeting places, are spaces, like home, where you can meet people, as in a family setting [40], where relations are based on equality and person-centredness. However, the mandatory documentation of healthcare within recovery-oriented Lts raises some questions. We are curious about how to document healthcare in such a setting, and whether it is natural to document it in an everyday life setting.

The participants in this study have used pictures and text to show and document what they experience as mental healthcare in a low threshold context, and to point out how healthcare appears in this context. Our contribution is to elaborate on the understanding of healthcare within recovery-oriented low threshold contexts. It is challenging to connect (today’s) medical understanding of healthcare, and a relational, recovery-oriented understanding of healthcare within the services as by definition they are contradictory, moving from patient-centred help in the medical understanding, to person-centred help within the recovery-oriented understanding. By using the traditional (medical) approach to healthcare in low threshold services, the “patient status” returns.

When it comes to the definition of healthcare in the legislation, our findings reveal several ticks in the boxes on the internal-control checklist [33] for what is considered to be healthcare. According to this checklist, all attendance at the Lts might be healthcare, based on the mandatory mapping during the first visit, and the fact that healthcare professionals are available to contribute support. This guides the way healthcare is documented within Lts and describes the everyday life context for many people struggling with mental health challenges.

The core dilemma points in the direction of whether it is possible to avoid assigning people patient status in their everyday life settings in a context focusing on their resources. If not, is it possible to find ways to accommodate the mandatory documentation of healthcare in the legislation based on recovery-oriented values?

Our findings show availability to be the main healthcare provider within low threshold services. Availability of people, places and activities is experienced as healthcare by service users in a low threshold context if they occur together simultaneously. This refers to the overall framework of Lts.

Our findings have shown there is a need for a reflexive practice with respect to healthcare in a low threshold context. The Lts framework is both legal and physical. Our findings have shown the relations, the physical framework and activities providing meaning-making processes to serve as experienced healthcare for the service users. Is it possible to use a wide lens, picturing the future where the legal obligations and recommendations according to recovery-oriented healthcare all fit in the same picture, the picture of healthcare?

We suggest a discussion on the healthcare definition. Our findings might contribute to this and to the development of the understanding of healthcare within low threshold contexts.

Future avenues of research should reflect on whether it is natural to document an everyday life setting, and if so, how to document healthcare based on the new or extended understanding of it.

## Data Availability

The generated photos from the photovoice-workshops, analysed during this study are included in this article. The rest of the datasets generated and analysed during the current study are not publicly available due to ethical approval.

## Abbreviation

Lts: Low threshold services
